# Chemical Composition and Biological Activities of Fragrant Mexican Copal (*Bursera* spp.)

**DOI:** 10.3390/molecules201219849

**Published:** 2015-12-12

**Authors:** Giulia Gigliarelli, Judith X. Becerra, Massimo Curini, Maria Carla Marcotullio

**Affiliations:** 1 Department of Pharmaceutical Sciences, University of Perugia, Via del Liceo, 1-06123 Perugia, Italy; giulygiglia@hotmail.it (G.G.); massimo.curini@unipg.it (M.C.); 2 Department of Biosphere 2, University of Arizona, Tucson, AZ 85721, USA; jxb@email.arizona.edu

**Keywords:** copal, *Bursera*, essential oil, terpenoids, resin, lignans

## Abstract

Copal is the Spanish word used to describe aromatic resins from several genera of plants. Mexican copal derives from several *Bursera* spp., *Protium copal*, some *Pinus* spp. (e.g., *P. pseudostrobus*) and a few *Fabaceae* spp. It has been used for centuries as incense for religious ceremonies, as a food preservative, and as a treatment for several illnesses. The aim of this review is to analyze the chemical composition and biological activity of commercial Mexican *Bursera* copal.

## 1. Introduction

The term ”resin” is often used to describe fragrant plant saps or exudates distinguished from other plant exudates such as gums, mucilages, oils, waxes, and latex. Plant resin is defined primarily as “a lipid-soluble mixture of volatile and non-volatile terpenoid, and/or phenolic secondary compounds that are (a) usually secreted in specialized structures located either internally or on the surface of the plant and (b) of potential significance in ecological interactions” [1,2]. Resins usually consist of a volatile fragrant fraction, usually called essential oil, and a non-volatile fraction, usually consisting of long-chain terpenoids. When fresh resins are translucent liquids but with time and the loss of the essential oil fraction, they turn into brown, yellow, or white solids that, by polymerization and oxidation, fossilize as amber [3].

Resins have been used since ancient times as constituents of varnishes, cosmetics, adhesives, and as incense in ritual ceremonies in temples and churches. Resins from three important genera of the Burseraceae—*Boswellia*, *Commiphora*, and *Bursera*—have been, and still are, used in perfumery and particularly as incense. *Boswellia* resin is called *frankincense*, *Commiphora* resin is commonly known as *myrrh* and *Bursera* resin is often referred to as *copal*.

The word copal derives from *copalli*, the Náhuatl (Atzec) term for incense. The Maya, in turn, used the term *pom* [4,5] for the incense derived from *Protium*, *Bursera*, and *Pinus*, depending on which resin-producing trees were most abundant in the areas where they lived. Later, the Spanish exported the term *copal* to Europe [1]. Nowadays, outside Mexico, the term is used for resins of the Fabaceae family and, generically, resins from Burseraceae are sometimes called *elemi* [6]. In Mexico and Guatemala copal derives mostly from *Bursera*, from a species of *Protium* (*Protium copal*) and a pine species (*Pinus pseudostrobus*) [3]. *Bursera*’s distribution encompasses tropical regions from southern United States (southern Arizona, California, and Florida) to Peru. In Mexico, the genus is highly diverse and abundant along the Pacific slopes [1]. *Protium copal* and *Pinus pseudostrobus* are found in Mexico and Central America.

In marketplaces of Central Mexico it is possible to find a variety of copal types to satisfy many tastes (and budgets, as prices vary according to the quality): *copal blanco*, *copal oro*, *copal negro*, *copal lágrima*, *copal incienso*, *copal de piedra* [3,6]. Several authors have analyzed different commercial copals and found great differences in the chemical composition among samples of the same type of copal, suggesting that the same name might currently be applied to products produced from several different plant species [3,6]. Furthermore, Case *et al*. [6] stated that different types of copal are derived from different collecting procedures. *Copal blanco* is the most common, being exuded directly from incisions made in the bark. *Copal oro* is from resin that is exuded after removal of the bark. *Copal negro* is beaten from bark [3] and *copal de piedra* is exuded as a defensive reaction to the attack of insects such as the Cerambycid beetle, *Chyptodes dejeani* [7]. *Copal lágrima* (copal in tears) is the product remaining in the recipient of collection and the incision in the bark [8]. Interestingly, Stacey *et al.* [6] found that a commercial sample of *copal lágrima* from the market of Tepoztlán, Morelos in central Mexico contained boswellic acids, commonly found in *Boswellia* (another Burseraceae genus not found in Mexico).

*Bursera* Jacq. ex L. (family Burseraceae, order Sapindales), a monophyletic genus [9] that consists of about 105 species, is a dominant taxon in seasonally dry tropical forests and also abundant in the deserts and oak savannahs of southern Mexico (*ca.* 85 species). It has been divided into two subgenera, *B*. subg. *Bursera* and *B.* subg. *Elaphrium*. Species of subgenus *Bursera* have 3–5-merous flowers, bivalvate fruits, and bark that typically exfoliates in colorful papery sheets or flakes, a trait that is responsible for their Aztec-derived name “cuajiote”, meaning *leprous tree*. Subgenus *Elaphrium* is characterized by a non-peeling grey-reddish rough bark, tetramerous flowers, and trivalvate fruits [10].

Regional variation in which *Bursera* species occur, as well as confusion in the literature about the species involved, has resulted in misunderstandings about which species are utilized as copal. Current efforts to disentangle this confusion include the use of chemical analytical methods to match compounds found in copal with the species that are the sources of the resin. While this is a promising method for recently collected copal, older copal samples will continue to be challenging to link to a species source. As collected resins harden, they gradually lose the volatile components that provide the main chemical distinctiveness to *Bursera* species, leaving the non-volatile elements that are more species-invariant [6]. This was explicit in results of De la Cruz-Cañizares *et al*. [11] who examined a “fresh” sample of Mexican copal (*Bursera cuneata*) from a supplier of artists’ materials (Casa Sierra, Mexico DF, Mexico) and a five-year old sample from a Sonora market (Sonora, Mexico; Table 1). They found considerable differences between the fresh and the five-year-old samples.

The aim of this paper is to review the chemical composition of resins from *Bursera* species used and marketed in Mexico as *copal*. Analytical data were collected from peer-reviewed papers found using SciFinder, Scopus and PubMed databases. Synonyms for *Bursera* species are those reported by Espinosa [12]. Other synonyms have been found on The Plant List web site [13]. Unless otherwise specified, common names are those reported in the “Excel” file found on the CONABIO web page [14].

**Table 1:** Comparison of a fresh sample of *Bursera cuneata* copal (C), a five-year-old sample (D), and samples given two different artificial treatments (sample C.1, prepared by dissolving resin in turpentine and sample C.2, obtained heating the resin at 100 °C for 10 min).

Compound ^a^	C	C.1	C.2	D
Verbenene	v ^b^			
*o*-Cymene	v	v	v	v
α-Pinene	v			v
Camphene	v			v
β-Pinene	v			v
α-Phellandrene	v			v
α-Terpinene	v			v
Limonene	v	v	v	v
γ-Terpinene	v		v	v
α-Terpinolene	v	v	v	v
Verbenone	v			
Carvacrol	v			
Sabinol	v			
4-Terpineol	v			v
Carvacrol methyl ether	v			
Fenchyl acetate	v			
*cis*-Calamenene	v			
Isoledene	v			
*trans*-Caryophyllene				v
Hexanedioic acid, bis(2-ethylhexyl) ester				v

^a^: Components are listed in order of their elution from a HP-5MS column [11]. ^b^: Presence of the compound in the resin, no amount reported [11].

## 2. Copal Species, Distribution and Composition

Linares and Bye [15] described extensively how “copaleros” collect the resin. They use a particular knife (quixala or quichala) to make incisions into the bark. They put a leaf of *Quercus glaucoides* under the cut to isolate the resin from impurities on the bark. To collect the liquid resin, they employ leaves of *Agave angustifolia*. They collect copal from July to September every year. To avoid killing or damaging the trees, resin is only collected from the same tree every two or three years [15]. These authors reported that the most appreciated species are *B. bipinnata* (Sessè & Moc. ex DC.) Engl. and *B. copallifera* (Sessè & Moc. ex DC.) Bullock. Nowadays, painters use *copal* as a binding medium for paint together with linseed oil.

Most of the phytochemical studies to identify the species used as *copal* have been done on commercial samples and on archeological Aztec objects [6,16]. “Fresh” resins have a characteristic pine-lemony smell due to volatile terpenes and alkanes such as α-pinene, β-phellandrene, limonene, δ-carene, and heptane [17], while “aged” resins are studied for the triterpenoidic composition of the non-volatile fraction [11]. *Pinus* resins, are characterized by a large volatile fraction (20%–50%) with monoterpenes predominating over sesquiterpenes while in the non-volatile component diterpene acids with abietane, pimarane, and labdane frameworks are common. Burseraceae resins contain mono- and sesquiterpenes in the volatile fraction and triterpenoids in the non-volatile fraction [1]. Particularly, *Protium* spp. terpenoids are dominated by α- and β-amyrin and *Bursera* spp. terpenoids contain lupane compounds (e.g., lupeol) [16]. Often the non-volatile fraction of *Bursera* spp. contains lignans.

### 2.1. Distribution, Synonyms, Common Names, and Primary Essential Oils of Described Bursera Species

A diversity of *Bursera* species are known to be used as incense, but only a small number are reported by several authors as commercial copal, specifically, *B. linanoe*, *B. copallifera*, *B. bipinnata*, and *B. fagaroides.* Other important incense sources that are not as commercialized are *B. microphylla, B. penicillata,* *B. simaruba, B. schelechtendalii,* and *B. excelsa.*

*Bursera bipinnata* (Sessè & Moc. ex DC.) Engl. (synonym: *Amyris bipinnata* [18], subg. *Elaphrium*) is commonly known as *copal cimarrón* and *copal santo*. It is distributed from southern Chihuahua and Sinaloa to Morelos, Guerrero, Oaxaca, and Chiapas. Although chemically variable, the main volatile component of its fresh resin is α-pinene (Becerra, J.X., Personal observations. 2015).

*Bursera copallifera* (Sessè & Moc. ex DC.) Bullock (synonyms: *B. jorullensis*, *Bullockia jorullensis*, *B. palmeri* var. *glabrescens*, *Elaphrium copalliferum* and *E. jorullense* [14], subg. *Elaphrium*) is commonly known as *copal ancho* but also *c'uájtsutacu* (Tarasco name) and *copalcuáuitl-patlahoac* (Náhuatl name) [12,19]. It is native to the dry forests from the Mexican states from Nayarit to Oaxaca and Puebla at altitudes of between 1000 and 1900 m. Its essential oil is rich in germacrene D and α-humulene [20].

*Bursera cuneata* (Schltdl.) Engl. (synonyms: *Elaphrium cuneatum* [14], subg. *Elaphrium*) is commonly known as *copal*, *copalillo*, *cuerecatzundi*, *cuerica-tzunda*, *cuiricatzunda* (Purépecha name) [18]. It is native to the Mexican oak-tropical deciduous forest transition zone from Jalisco to Oaxaca. Its essential oil is relatively abundant in α-pinene, β-caryophyllene, and germacrene D (Becerra, J.X., Personal observations. 2015).

*Bursera excelsa* (Kunth) Engl. (synonyms: *Bullockia sphaerocarpa* and *Elaphrium excelsum* [14], subg. *Elaphrium*) is commonly known as *tecomahaca* and *copalquín* in Náhuatl language. It is largely present along the pacific coast of Mexico (Nayarit, Chiapas, Jalisco, Durango, *etc.*). Its fresh resin is rich in germacrene D and β-caryophyllene [20].

*Bursera fagaroides* (H.B.K.) Engl., or “fragrant bursera” (synonyms *B. obovata, B. schaffneri* [21], subg. *Bursera*) exists in three different varieties: *elongata*, *fagaroides*, and *purpusii* [22]. CONABIO and The Plant List database report several synonyms, such as *B. lonchophylla, B. tenuifolia*, *B. schafferii*, *Elaphrium* *covillei*, *E. inaguense*, *Amyris ventricosa* [13,14]. It is commonly known as *aceitillo*, *copa*, *cuajiote amarillo* and *jiote* (Náhuatl name) [14]. It is native to northern Mexico, (Sinaloa, Sonora) and the central and southern states (Queretaro, Guerrero, Jalisco, Michoacan, Nayarit, Oaxaca, *etc.*). The volatile chemistry of this taxon is highly variable, but plants often contain large amounts of α-pinene, β-phelandrene, germacrene B, and germacrene D (Becerra, J.X., Personal observations. 2015). Copal studies on *B. fagaroides* most often do not identify variety investigated. 

*Bursera linanoe* (La Llave) Rzed., Calderón & Medina (synonyms: *B. aloexylon*, *B. delpechiana*, *B. longipedunculata*, *Amyris* *linaloe*, *Elaphrium longipedunculatum* [13], subg. *Elaphrium*), also known as Indian lavender tree. This is one of the species most extensively used as copal by the indigenous Mexican people in the past as well as in the present. The XVI century Spanish historian Francisco Hernandez describes this species known to the Aztecs as “*Copalcuáuitl*”, meaning *copalli* *tree*, now commonly known as *copal blanco* [19]. Their drawings of the plant source of this copal also closely resemble live *B. linanoe* trees, confirming its identity. This species produces one of the most pleasant and fragrant resins and is currently cultivated in India for use in the perfume industry [23]. It is the only *Bursera* species whose essential oil consists predominantly of linalyl acetate [23,24].

*Bursera microphylla* A. Gray (synonyms: *Elaphrium microphyllum*, *Terebinthus microphylla*, subg. *Bursera*) is commonly known as elephant tree, *torote*, *torote blanco*, *copal*, or *cuajiote colorado* and is native to the Sonoran Desert, from southwestern Arizona and southeastern California, to the western Mexican mainland, and Baja California [16,25,26]. The chemistry of this species also varies greatly among geographic locations. The resin acetonic extracts of different samples from two different populations (Guaymas and La Paz) were studied by Mooney and Emboden [27] who identified α-pinene, β-pinene, phellandrene, limonene, cineole, and four unidentified compounds, while Tucker and coll. [28] found that plant samples from Southern Arizona were rich in β-caryophyllene.

*Bursera penicillata* (Sessè & Moc. ex DC.) Engl. (synonyms: *Amyris penicillata*, *Bursera mexicana*, *Elaphrium delpechianum*, *E. mexicanum*, *E. penicillatum*, *Terebinthus delpechiana*, *T. mexicana* [13,14], subg. *Elaphrium*) is also known as *Bullockia inopinata* [14]. Its common name is *torote incienso* and *torote copal*. It is native to the westerns states of Sonora, Aguascalientes, Sinaloa, Nayarit, Zacatecas, Michoacan, *etc.* [21].

*Bursera schlechtendalii* Engl. (synonyms: *B. jonesii*, *E. jonesii*, *Terebinthus jonesii*, *T. schlechtendalii*, subg. *Bursera*). It is native to Central Mexico and Guatemala. Its fresh resin contains large amounts of the highly volatile heptane, β-phellandrene, sabinene, nonane, and myrcene [29].

*Bursera simaruba* (L.) Sarg. (synonyms: *B. bonairensis*, *B. gummifera*, *B. integerrima*, *B. subpubescens*, *Elaphrium arboretum*, *E. integerrimum*, *E. simaruba*, *Terebinthus simaruba*, *T. arborea* [13], subg. *Bursera*) is commonly known as *chacaj* or *chakaj* (Tojolabal name), *yaga-guito* (Zapotec name) [14]. It has a wide distribution in Mexico and Central America. Its volatile chemistry varies among locations, but it contains α-pinene, β-pinene, and a diversity of sesquiterpenoids including α-copaene, δ-elemene, β-caryophyllene, germacrene D, germacrene B, and β-sesquiphellandrene (Becerra, J.X., Personal observations. 2015).

### 2.2. Composition of the Triterpenoid Fraction

**Figure 1:**
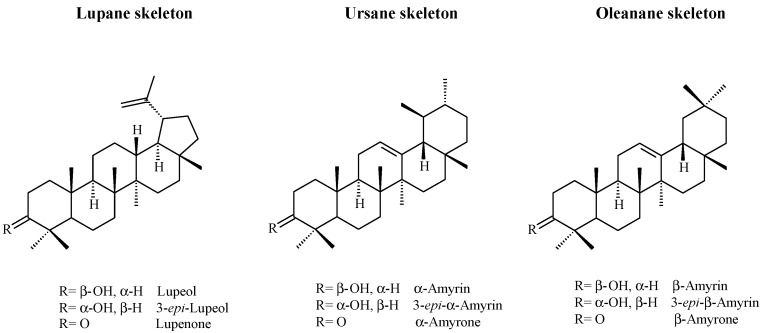
Triterpenoid markers used in GC-MS analysis of copal [16].

Triterpenoid of the lupane type are characteristic of *Bursera* resins, but often ursane and oleanane triterpenoids are also present (Figure 1). Stacey *et al.* [6] examined and compared *copal* resins from different ancient artefacts from the British Museum, botanical specimens from *Pinus*, *Protium* and *Bursera* spp. and commercial samples of *copal lágrima*, *copal negro*, *copal incienso*, *copal de piedra* and *copal blanco*, from the market of Tepoztlan, Morelos. They found that the samples of commercial *copal blanco*, *negro* and *de piedra* have similar terpenoid profiles characterized by 3-*epi*-β-amyrin, 3-*epi*-α-amyrin, lupeol and α-amyrin, similar to that of a fifty year old *B. excelsa* sample but with some affinity with *B. linanoe*. The fresh sample of *B. fagaroides* var. *fagaroides* that they examined showed a completely different profile, dominated by oleanonic and ursonic acids. Furthermore, as mentioned above, they found that the commercial sample of *copal lágrima* has a terpenoid composition resembling *Boswellia*, due to the presence of boswellic acids.

Lucero-Gómez *et al*. [30] studied the triterpenoid composition of fresh samples of different *Bursera* copals using GC, and comparing them to nine triterpene standards (3-*epi*-β-amyrin, 3-*epi*-α-amyrin, 3-*epi*-lupeol, β-amyrone, β-amyrin, α-amyrone, α-amyrin, lupenone, lupeol) derivatized as trimethylsilyl ethers (OTMS).

They analyzed *B. bipinnata*, *B. excelsa*, *B. copallifera* and *B. penicillata*, but also *B. stenophylla* as its botanical distinction from *B. bipinnata* is unclear [21], *B. simaruba* because it was used as binder in Bonampak murals (Maya) [16] and *B. grandifolia* because it is phylogenetically related to *B. simaruba*. In Table 2 we report the phytochemical results for these species. The authors found that the GC-MS profiles of *B. bipinnata* and *B. stenophylla* are identical.

**Table 2:** Non-volatile terpene fraction composition of *Bursera* resins studied by Lucero-Gómez [16].

Compound	*B. bipinnata* and *B. stenophylla*	*B. copallifera*	*B. excelsa*	*B. penicillata*	*B. grandifolia*	*B. simaruba*
3-*epi*-β-amyrin	v ^a^	= ^b^	v	v	v	v
3-*epi*-α-amyrin	v	=	v	v	v	v
3-*epi*-lupeol	v	=	v	v	v	v
β-amyrone	v	=	=	v	=	v
β-amyrin	v	=	=	v	v	v
α-amyrone	v	=	=	=	=	v
α-amyrin	v	=	=	v	v	v
lupenone	v	v	=	=	=	=
lupeol	v	v	=	v	=	v

^a^: Presence of the compound in the resin, no amount reported; ^b^: not found.

They identified in *B. bipinnata* all the nine standards and four more unidentified compounds (Table 2). Studying the triterpenic profile of *B. copallifera*, Lucero-Gómez *et al*. found lupeol and lupenone and five other molecules. From the fragmentation pattern in GC-MS analysis, they established the nature of four of them: three urs-12-ene derivatives and one olean-12-ene derivative [16]. In *B. excelsa* they identified only three of the nine standards: 3-*epi*-α- and 3-*epi*-β-amyrins and 3-*epi*-lupeol. Furthermore, they noted the presence of five other unidentified triterpenoids, three of which had MS fragmentation patterns similar to olean-12-ene type molecules and two of which were amyrones. In *B. penicillata*, they found a greater variety of triterpenoids than other resins. The only missing standards were α-amyrone and lupenone. Furthermore, they found seven other triterpenoids, three of which were identified as olean-12-ene derivatives (Table 2). In *B. simaruba* all of the standards were found except lupenone. Five other triterpenoids were detected in this resin, one of which was identified as an urs-12-ene compound. *B. simaruba* and *B. grandifolia* GC profiles are different, as is shown in Table 2.

The phytochemical composition of the non-volatile fraction of *B. microphylla* fresh resin collected near Hermosillo, Sonora was recently studied by our research group. The methanol extract of the resin was divided by means of solvent with different polarity into two sub-fractions: the hexane sub-fraction (H) and the dichloromethane one (DCM). The H-sub-fraction contained several terpenoids and lignans.

**Figure 2:**
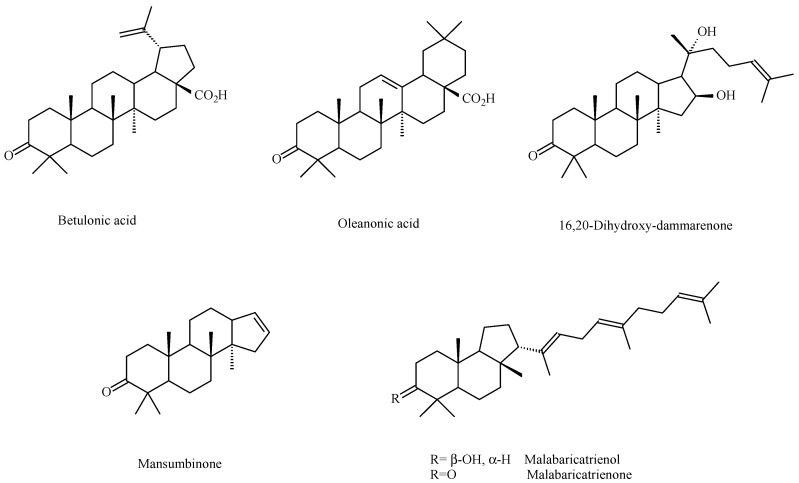
Triterpenoidic compounds isolated from *B. microphylla* [31].

**Figure 3:**
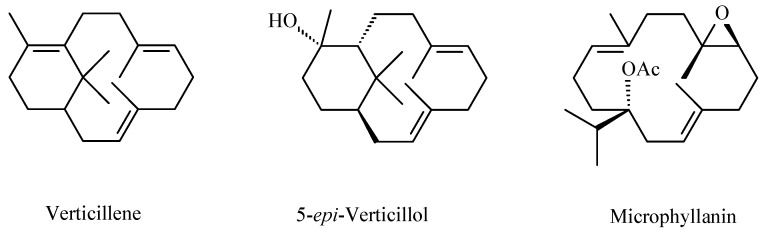
Diterpene compounds isolated from *B. microphylla* [31].

**Figure 4:**
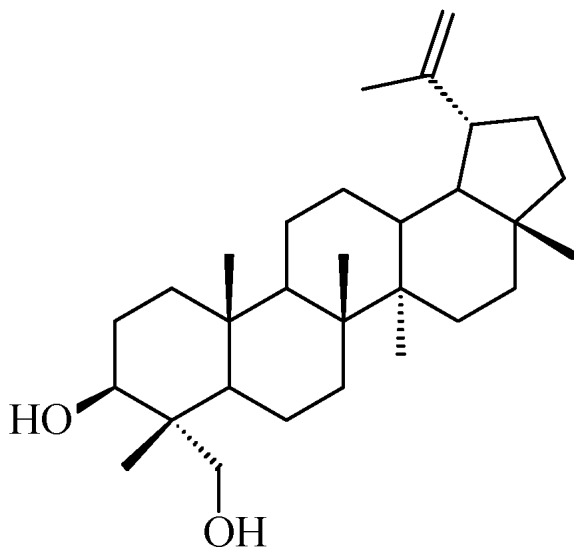
New lupane-type triterpene isolated from *B. simaruba* [33].

The presence of β-caryophyllene and caryophyllene oxide was confirmed. Known triterpenoids with a lupane (betulonic acid), oleanane (oleanonic acid), and dammarane (16,20-dihydroxy-dammarenone and mansumbinone) skeleton were isolated (Figure 2). Furthermore, two new triterpenoids with the quite rare malabaricane skeleton (malabaricatrienone and malabaricatrienol) were present (Figure 2). In the hexane subfraction there were several diterpenoids such as verticillene, 5-*epi*-ent-verticillol (verticillane skeleton) and microphyllanin (Figure 3) [31]. Microphyllanin is a cembrane diterpenoid, that has been previously isolated from *B. multijuga* [32]. Peraza-Sánchez *et al*. isolated in 1995 a new lupane-type triterpene from the chloroformic resin extract of *B. simaruba* (Figure 4) [33].

### 2.3. Composition of the Lignan Fraction

**Figure 5:**
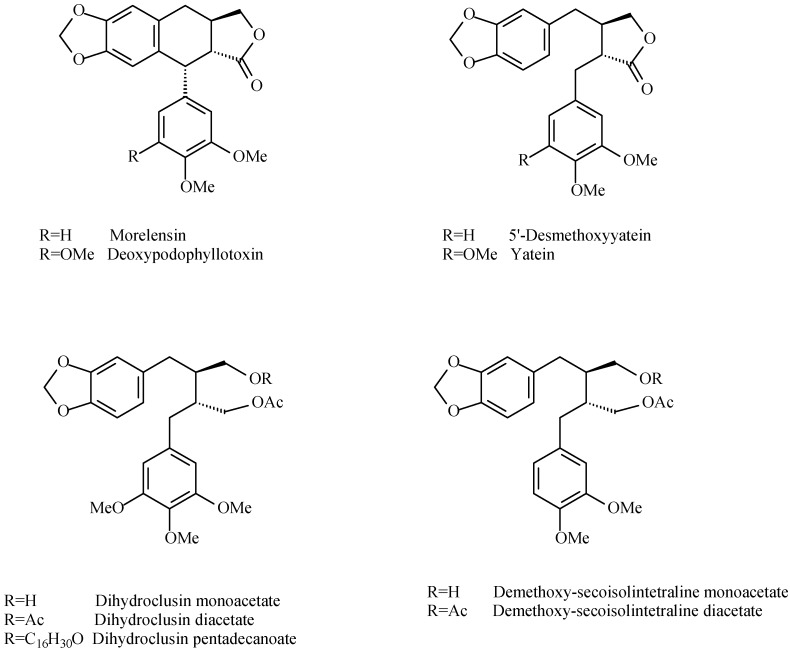
Lignans isolated from *B. fagaroides* resin [37,38].

Lignans are phenolic components of foods and medicines that arise from radical coupling of two units of coniferyl alcohol. Lignans can be classified into different groups based on skeleton oxidation and functionalization [34,35]. *B. simaruba*, *B. fagaroides* and *B. microphylla* exudates have been studied for lignan content. Most of the studies report the lignan content of bark, stem or leaves extracts [36]. Velazquez-Jimenez *et al*. [37] isolated from *B. fagaroides* resin two aryltetraline lignans ((−)-deoxy-podophyllotoxin, (−)-morelensin) and two dibenzylbutirolactone lignans ((−)-yatein and (−)-5′-des-methoxyyatein). The authors determined the absolute configuration of these compounds by comparison of the vibrational circular dichroism spectra of known podophyllotoxin and desoxypodophyllotoxin with those obtained by density functional theory calculations. Other diarylbutane lignans were isolated by Morales-Serna *et al*. [38] from the chloroform extract of *B. fagaroides* resin: 9-acetyl-9′-pentadecanoyl-dihydroclusin, 2,3-demethoxy-secoisolintetralin monoacetate, dihydroclusin monoacetate, together with previously known 2,3-demethoxysecoisolintetralin diacetate and dihydroclusin diacetate (Figure 5).

**Figure 6:**
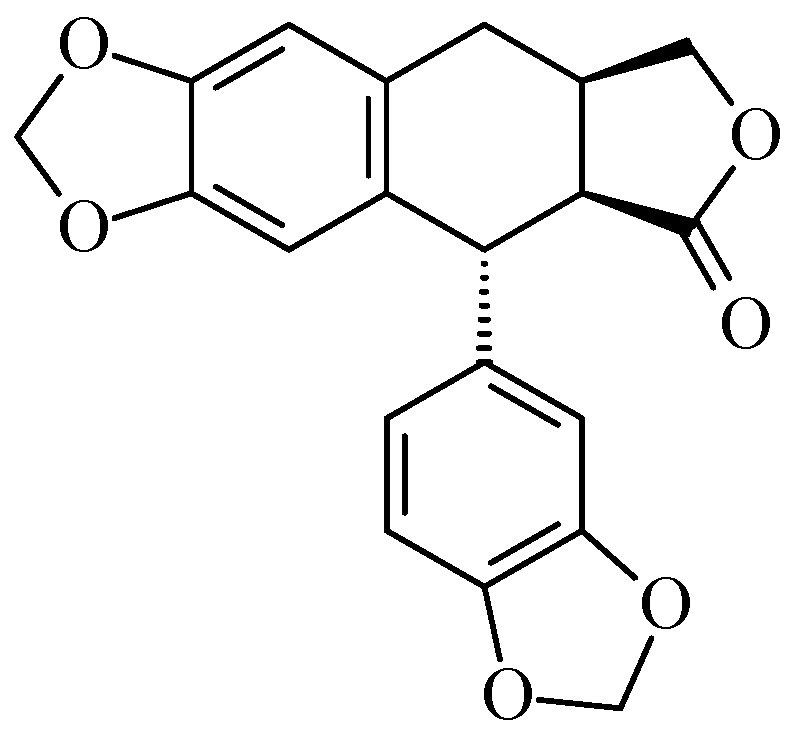
Picropolygamain isolated from *B. simaruba* [40].

Morelensin was isolated for the first time by Jolad *et al*. from dried exudate of *Bursera morelensis* [39]. Peraza-Sánchez *et al.* isolated for the first time picropolygamain from the chloroformic extract of *B. simaruba* resin (Figure 6) [40].

**Figure 7:**
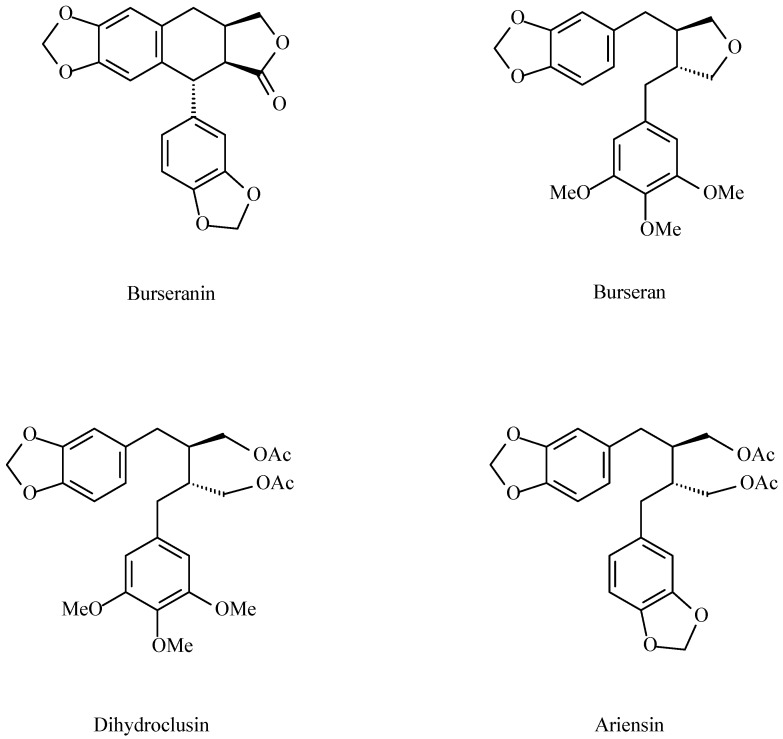
Lignans isolated from *B. microphylla* resin [31].

The phytochemical analysis of *B. microphylla* hexane subfraction of a methanolic extract, led to the isolation of four known lignans: burseranin, burseran, ariensin and dihydroclusin diacetate [31] (Figure 7).

**Figure 8:**
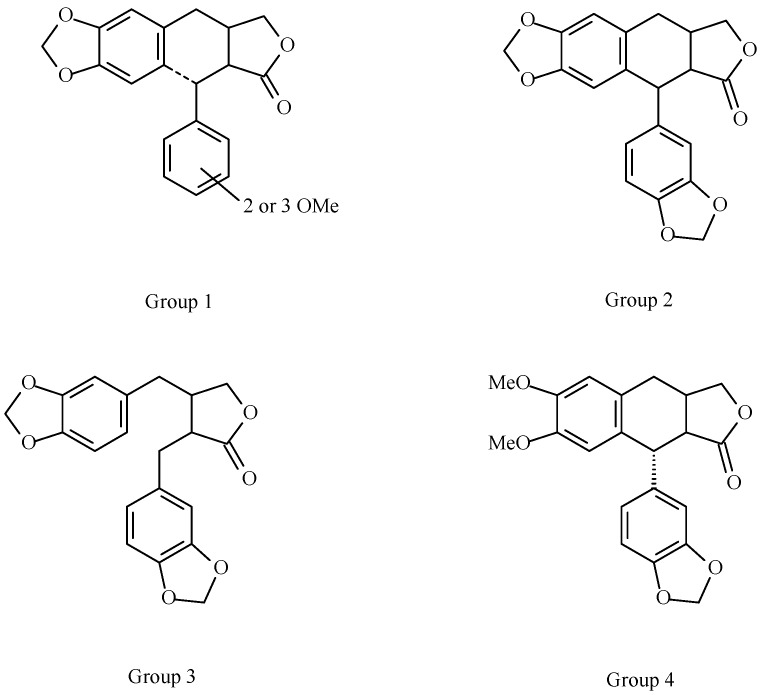
Groups of lignans present in *Bursera* spp. leaves according to Koulman [36].

It is interesting to note that lignans isolated from copal, with the exception of burseran, belong to the aryltetraline and dibenzylbutane groups. Koulman studied the biosynthesis of *Bursera* lignans from matairesinol and he classified them in four different groups (Figure 8) [36]. From his studies on dry leaves, Koulman noted that groups 1 and 2 lignans are present in subg. *Bursera* (*B. fagaroides* and *B. microphylla*) while groups 3 and 4 are in subg. *Elaphrium* (*B. bipinnata*, *B. copallifera*, *B. cuneata*, *B.* *excelsa* and *B. penicillata*). The few studies on lignans isolated from *Bursera* resins, are in agreement with these results. Lactone lignans isolated from *B. fagaroides* and *B. microphylla* (both in subg *Bursera*) belong to group 1.

### 2.4. Biological Activities of Copal.

Case and Orta-Amaro [3,41] described many of the ancient and traditional uses of copal. Mesoamerican people used copal for different purposes. First of all, it was considered to be food for the gods, but it was also used as incense used during ceremonies, as a binder mixed with pigments for painting, for the decoration of murals and for the preparation of holy artifact. “Copal served as the “flesh” of idols with a wooden skeleton that were further covered with a rubber skin” [3]. Copal smoke was used to cure headache and to clean the body after being exposed to sick people [19]. Copal ground and dissolved in water was used by the Nahua people to treat diarrhea, as an anti-inflammatory poultice [41], to plug tooth cavities (Nahua and Maya), and to treat pneumonia. *Bursera copallifera* was used against uterine diseases. *Bursera bipinnata* has been used to treat wounds and *B. simaruba* has been used to treat fever and chicken pox [3]. The Seri Pharmacopoeia reports the use of *B. microphylla* resin for sore throats, headache, and for wound healing [42]. In Oaxaca city, copal of *B.* *fagaroides* is used to prepare infusions to treat stomach problems and as an anti-inflammatory [38].

Copal was, and still is, primarily used as folk medicine and only a few scientific papers are found in the literature that have investigated its biological activity. *Bursera bipinnata* copal was studied for its film-forming potential and its potential use as coating material for sustained release and colon-targeted drug delivery [43]. Velazquez-Jimenez *et al*. prepared an ethanol extract of the dry exudate of *B. fagaroides* and found it cytotoxic, in a concentration-dependent manner, against HT-29 (human colorectal adenocarcinoma) cells with IC_50_ values of 0.40 and 0.41 μg/mL after 48 and 72 h, respectively [37]. As already mentioned, these authors isolated from this extract four podophyllotoxin related lignans from this extract [37]. The methanol extract of *B. microphylla* was studied for its cytotoxic activity against human cancer cell lines: A549 (lung cancer) (IC_50_ 53.77 μg/mL) andHeLa (cervix cancer) (IC_50_ 13.85 μg/mL), and against the murine cell lines M12.C3.F6 (B cell lymphoma) (IC_50_ 26.00 μg/mL). Following these encouraging preliminary results, the new and the already known compounds isolated by hexane fraction were tested for their cytotoxic activity against three human cancer cell lines, namely, A549, HeLa, and PC-3 (prostate cancer), and against the murine cell lines M12.C3.F6 and RAW264.7 (macrophages transformed by the virus Abelson leukemia). Malabricatrienone, malabaricatrienol and microphyllanin (Figure 2 and Figure 3) were found to be inactive, and among the known compounds, only dihydroclusin diacetate was shown to be active against murine cell line M12.C3.F3 (IC_50_ 2.5 μM), while ariensin, burseran, and dihydroclusin diacetate (Figure 5) were active against the RAW246.7 murine cell line (IC_50_ 9.8, 0.4, and 0.2 μM, respectively). Betulonic acid (Figure 2) was shown to be active against all the tested lines (IC_50_: M12.C3.F3 = 13.2 μM, A549 = 12.6 mM, HeLa = 13.6 μM, RAW 264.7 = 10.2 μM, PC-3 = 18.6 μM) [31].

Although few studies have been reported on the biological activities of *Bursera* copal, several of the isolated compounds have been studied. Many terpenoids and lignans isolated from *Bursera* copal have been studied and several reviews on their biological activity have been published. For example biological properties of lupeol, α- and β-amyrins and lignans have been recently reviewed [44,45,46].

## 3. Conclusions

Our analysis of the literature showed that in Mesoamerica the term “copal” currently does not have an unequivocal botanical association and that, despite continued widespread use, few data are available on the analytical composition of these resins. Due to the extensive studies of historical artifacts, most of the research efforts have been conducted on the triterpenoid fraction, while a limited number of studies are reported about the volatile fraction composition. Furthermore, deeper studies have to be made to validate the biological and pharmacological properties of these resins that are commonly used in ethnopharmacology.

## References

[1] Langenheim J.H. (2003). Plant Resins: Chemistry, Evolution, Ecology and Ethnobotany.

[2] Becerra J.X. (1994). Squirt-gun defense in *Bursera* and the chrysomelid counterploy. Ecology.

[3] Case R.J., Tucker A.O., Maciarello M.J., Wheeler K.A. (2003). Chemistry and ethnobotany of commercial incense copals, copal blanco, copal oro, and copal negro, of North America. Econ. Bot..

[4] Barrera Marín A., Barrera Vásquez A., López Franco R.M. (1976). Nomenclatura Etnobotánica Maya: UNA Interpretación Taxonómica.

[5] McGee R.J. (1990). Life, Ritual, and Religion among the Lacandon Maya.

[6] Stacey R.J., Cartwright C.R., McEwan C. (2006). Chemical characterization of ancient mesoamerican “copal” resins: preliminary results. Archaeometry.

[7] Montúfar A. (2007). Los Copales Mexicanos y la Resina Sagrada del Templo Mayor de Tenochtitlan.

[8] Cruz León A., Salazar Martínez L., Campos Osorno M. (2006). Antecedentes y actualidad del aprovechamiento de copal en la Sierra de Huautla, Morelos. Rev. Geogr. Agric..

[9] Becerra J.X., Noge K., Olivier S., Venable D.L. (2012). The monophyly of *Bursera* and its impact for divergence times of Burseraceae. Taxon.

[10] Becerra J.X., Venable D.L. (1999). Nuclear ribosomal DNA phylogeny and its implications for evolutionary trends in Mexican *Bursera* (Burseraceae). Am. J. Bot..

[11] De la Cruz-Cañizares J., Doménech-Carbó M.-T., Gimeno-Adelantado J.-V., Mateo-Castro R., Bosch-Reig F. (2005). Study of burseraceae resins used in binding media and varnishes from artworks by gas chromatography-mass spectrometry and pyrolysis-gas chromatography-mass spectrometry. J. Chromat. A.

[12] Espinosa D. (2007). Catálogo de Autoridades Taxonómicas de Las Burseráceas (Burseraceae: Magnoliopsida) de México.

[13] ThePlantList A Working List of All Plant Species. http://www.theplantlist.org/1.1/browse/A/Burseraceae/Bursera/.

[14] CONABIO Burseraceae. http://www.conabio.gob.mx/informacion/catalogo_autoridades/doctos/bruseras.html.

[15] Linares E., Bye R. (2008). El Copal en Mexico. Biodiversitas.

[16] Lucero-Gómez P., Mathe C., Vieillescazes C., Bucio L., Belio I., Vega R. (2014). Analysis of mexican reference standards for *Bursera* spp. resins by gas chromatography-mass spectrometry and application to archaeological objects. J. Archeol. Sci..

[17] Becerra J.X., Venable D.L., Evans P.H., Bowers W.S. (2001). Interactions between chemical and mechanical defenses in the plant genus *Bursera* and their implications for herbivores. Am. Zool..

[18] Rzedowski J., Guevara-Féfer F. (1992). Burseraceae. Flora del Bajío y de Regiones Adyacentes.

[19] Hernandez F., Ochoterena I. (1943). Historia de Las Plantas de Nueva Espana.

[20] Noge K., Becerra J.X. (2009). Germacrene D, a common sesquiterpene in the genus Bursera (Burseraceae). Molecules.

[21] Rzedowski J., Medina Lemos R., Calderón de Rzedowski G. (2005). Inventario del conocimiento taxonómico, así como de la diversidad y del endemismo regionales de las especies mexicanas de *Bursera* (Burseraceae). Acta Bot. Mex..

[22] McVaugh R., Rzedowski J. (1965). Synopsis of the genus *Bursera* L. in western Mexico, with notes on the material of *Bursera* collected by Sessè & Mocino. Kew Bull..

[23] Becerra J.X., Noge K. (2010). The Mexican roots of the Indian lavender tree. Acta Bot. Mex..

[24] Noge K., Shimizu N., Becerra J.X. (2010). (R)-(−)-linalyl acetate and (S)-(−)-germacrene D from the leaves of Mexican *Bursera linanoe*. Nat. Prod. Commun..

[25] Felger R.S., Johnson M.B., Wilson M.F. (2001). The Trees of Sonora, Mexico.

[26] Turner R.M., Bowers J.E., Burgess T.L. (2005). Sonoran Desert Plants: An Ecological Atlas.

[27] Mooney H.A., Emboden W.A. (1968). The relationship of terpene composition, morphology, and distribution of populations of *Bursera microphylla* (Burseraceae). Brittonia.

[28] Tucker A.O., Maciarello M.J., Brown R.C., Landrum L.R., Lafferty D. (2009). Essential oils from the oleo-gum-resins of elephant tree or Torote (*Bursera microphylla* A. Gray, Burseraceae) from Arizona. J. Essent. Oil Res..

[29] Evans P.H., Becerra J.X., Venable D.L. (2000). Chemical analysis of the squirt-gun defense in *Bursera* and the counterdefense by chrysomelid beetles. J. Chem. Ecol..

[30] Lucero-Gómez P., Mathe C., Vieillescazes C., Bucio-Galindo L., Belio-Reyes I., Vega-Aviña R. (2014). HPLC: Molecular profiles for the discrimination of copals from Mesoamerica. Application to objects from Aztec offerings. J. Archaeom..

[31] Messina F., Curini M., di Sano C., Zadra C., Gigliarelli G., Rascon-Valenzuela L.A., Robles Zepeda R.E., Marcotullio M.C. (2015). Diterpenoids and triterpenoids from the resin of *Bursera microphylla* and their cytotoxic activity. J. Nat. Prod..

[32] Hernandez-Hernandez J.D., Garcia-Gutierrez H.A., Roman-Marin L.U., Torres-Blanco Y.I., Cerda-Garcia-Rojas C.M., Joseph-Nathan P. (2014). Absolute configuration of cembrane diterpenoids from *Bursera multijuga*. Nat. Prod. Commun..

[33] Peraza-Sánchez S.R., Salazar-Aguilar N.E., Peña-Rodríguez L.M. (1995). A new triterpene from the resin of *Bursera simaruba*. J. Nat. Prod..

[34] Moss G.P. (2000). Nomenclature of lignans and neolignan. Pure Appl.Chem..

[35] Suzuki S., Umezawa T. (2007). Biosynthesis of lignans and norlignans. J. Wood Sci..

[36] Koulman A. (2003). Podophyllotoxin: A Study of the Biosynthesis, Evolution, Function and Use of Podophyllotoxin and Related Lignans. Ph.D. Thesis.

[37] Velázquez-Jiménez R., Torres-Valencia J.M., Cerda-García-Rojas C.M., Hernández-Hernández J.D., Román-Marín L.U., Manríquez-Torres J.J., Gómez-Hurtado M.A., Valdez-Calderón A., Motilva V., García-Mauriño S. (2011). Absolute configuration of podophyllotoxin related lignans from *Bursera fagaroides* using vibrational circular dichroism. Phytochemistry.

[38] Morales-Serna J.A., Cruz-Galicia E., Garcia-Rios E., Madrigal D., Gavino R., Cardenas J., Salmon M. (2013). Three new diarylbutane lignans from the resin of *Bursera fagaroides*. Nat. Prod. Res..

[39] Jolad S.D., Wiedhopf R.M., Cole J.R. (1977). Cytotoxic agents from *Bursera morelensis* (Burseraceae): Deoxypodophyllotoxin and a new lignan, 5′-desmethoxydeoxypodophyllotoxin. J. Pharm. Sci..

[40] Peraza-Sánchez S.R., Peña-Rodríguez L.M. (1992). Isolation of picropolygamain from the resin of *Bursera simaruba*. J. Nat. Prod..

[41] Orta-Amaro M. (2007). Copal: Microestructura, Composición y Algunas Propiedades Relevantes. Ph.D. Thesis.

[42] Felger R.S., Moser M.B. (1973). Seri Indian pharmacopoeia. Econ. Bot..

[43] Sharma J., Kaur L., Kanuja N., Nagpal M., Bala R. (2013). Natural polymers-promising potential in drug delivery. Int. J. Pharm.Technol. Res..

[44] Siddique H.R., Saleem M. (2011). Beneficial health effects of lupeol triterpene: A review of preclinical studies. Life Sci..

[45] Hernández Vázquez L., Palazon J., Navarro-Ocaña A., Rao D.V. (2012). The pentacyclic triterpenes a,b-amyrins: A review of sources and biological activities. Phytochemicals—A Global Perspective of Their Role in Nutrition and Health.

[46] Cunha W.R., e Silva M.L.A., Veneziani R.C., Ambrósio S.R., Bastos J.K., Rao D.V. (2012). Lignans: Chemical and Biological Properties. Phytochemicals—A Global Perspective of their Role in Nutrition and Health.

